# A case report of acute food protein-induced enterocolitis syndrome to walnut

**DOI:** 10.1186/s13223-023-00812-4

**Published:** 2023-07-01

**Authors:** Wen Zheng Zhu, Siobhan Perkins

**Affiliations:** 1 Doctor of Medicine undergraduate medical program, University of British Columbia, Vancouver, Canada; 2 Division of Allergy and Immunology, Department of Medicine, University of British Columbia, Vancouver, BC Canada; 3 Division of Allergy and Immunology, Department of Internal Medicine, University of British Columbia, Vancouver, BC Canada

**Keywords:** FPIES, Food protein-induced enterocolitis syndrome, Walnut, Tree nuts, Late onset

## Abstract

**Background:**

Food protein-induced enterocolitis syndrome (FPIES) is a delayed, non-IgE-mediated food allergy. This syndrome was once thought to be rare, but emerging literature suggests an increasing incidence along with more foods being implicated. Also, with the introduction of guidelines on early peanut introduction, peanut-induced FPIES seems to be increasing in Australia and USA. Although most patients are diagnosed with FPIES within the first year of life, most commonly with food triggers to cow’s milk or soy, other phenotypes exist in comparison to this classic presentation. In this case report, we present a patient with late onset of acute FPIES at age 3 to walnut.

**Case Presentation:**

We describe a case of FPIES in a 12-year-old boy who had recurrent episodes of repetitive emesis, that began at age 3, each time after consuming walnuts. Mom does not give a history of intentional feeding (or avoidance) of walnut and/ or pecans. She also described possible reactions with pine nuts and macadamia. He was assessed with an oral food challenge to walnut, which triggered an episode of acute FPIES. He developed vomiting with onset 2 h post-ingestion, pallor, lethargy, and required an emergency department visit for anti-emetic medications and oral rehydration therapy. He improved on the therapy and now avoids cashew, pistachio, hazelnuts, walnuts, pecans, pine nuts, and macadamia nuts.

**Conclusions:**

This case report adds to the limited literature that exists on culprit food allergens in FPIES. We present an acute FPIES triggered by the ingestion of walnuts. The diagnosis, common food triggers, and natural history of FPIES are described. There remains a lack of information on the natural history of FPIES, especially towards uncommon food triggers and on FPIES that present beyond infancy.

## Background

Food protein-induced enterocolitis syndrome (FPIES) is a delayed, non-IgE-mediated food allergy. Its major feature is delayed emesis one to four hours after consumption of the triggering food. It is often associated with lethargy, pallor, and diarrhea. It can be difficult to recognize a reaction as FPIES due to the delay of symptoms in association with the triggering food.

FPIES was first described as a delayed enterocolitis illness by Powell in 1976 in two infants that developed a recurring syndrome of vomiting, bloody diarrhea, abdominal distension, septic appearance, and hypothermia after consumption of cow’s milk and soy-based formulas [1].

Since then, a limited number of epidemiologic studies have been conducted, with an incidence range between 0.015% and 0.7% [2–5]. In the United States, the prevalence rate has been estimated at 0.5% of all children [6]. These data give further support that FPIES may not be as rare as once thought, and evolving literature indicate rising incidence rates with more foods being implicated [7]. Furthermore, with the introduction of guidelines on early peanut introduction, peanut-induced FPIES seems to be increasing in Australia and USA [8, 9]. However, given challenges in diagnosis, the true incidence and prevalence of FPIES remain difficult to characterize, and is likely underdiagnosed. These challenges can be partly attributed to unfamiliarity with FPIES due to its relatively new recognition as a syndrome, varying diagnostic criteria and clinical presentations, and geographical differences in FPIES food triggers.

The diagnostic criteria for acute FPIES as defined by Nowak-Wegrzyn et al. in the international consensus guidelines is presented below (Table 1) [10]. The gold standard for diagnosis is with an oral food challenge (OFC) with the suspected food. Diagnosis is made clinically, as there are no confirmatory laboratory or imaging tests. Part of the diagnostic challenge with FPIES is that it can present with different phenotypes [10]. These include early versus late, acute versus chronic, mild-to-moderate versus severe, solid food FPIES, and atypical FPIES. In atypical FPIES, patients have positive serum IgE, which is associated with a more prolonged course of reactivity [11, 12].

**Table 1:** Diagnostic criteria for acute FPIES

Major criterion:	Minor criteria:
Repetitive emesis 1–4 h after consuming the suspected food, in the absence of classic IgE-mediated allergic skin or respiratory symptoms.	1. Repeated episodes of repetitive emesis after eating the same suspected food.
	2. Repetitive emesis 1–4 h after consuming a different food.
	3. Extreme lethargy.
	4. Pallor.
	5. Need for emergency department visit.
	6. Need for intravenous fluid support.
	7. Diarrhea within 24 h.
	8. Hypotension.
	9. Hypothermia.
To diagnose FPIES, patients must meet the major criterion and ≥ 3 minor criteria.	

Early versus late FPIES is defined based on age of onset, with the former being younger than 9 months old [10]. In acute FPIES, there is intermittent exposure to the food trigger, with resolution of symptoms within 24 h after eliminating the specific food. Chronic FPIES occurs during daily consumption of the food trigger, and is associated with chronic diarrhea, intermittent emesis, and poor weight gain. Once the food trigger is eliminated, symptoms resolve within 3–10 days. In mild-to-moderate FPIES, symptoms of emesis, diarrhea, pallor, and mild lethargy can be present. In severe cases, hospitalization may be required due to hypothermia, methemoglobinemia, acidemia, and hemodynamic instability. Although FPIES is generally first recognized in infancy once common triggers such as cow’s milk or soy products are introduced, FPIES secondary to solid foods are also seen. While cow’s milk and soy remain the most common food triggers, others such as grains, rice, meats, and fruits are some previously reported solid food triggers for FPIES [13].

There is limited literature on food triggers particularly to solid foods, and on patients diagnosed beyond infancy. We present a case report on acute FPIES in a 12-year-old patient, with a late onset of symptoms at age 3 to walnut.

## Case presentation

The patient is a 12-year-old boy with a history of mild to moderate episodic eczema that is well controlled on topical corticosteroids. He is also diagnosed with a seizure disorder and developmental delay and decreased communication ability. He was referred for re-assessment of multiple episodes of vomiting when he consumed walnut.

At age 3, he experienced two episodes of recurrent emesis four hours after consuming walnuts and at another time eating a cookie ( his mother is ensure if it contained a nut). On review of his initial assessment ( at the age of three), it is not clear if he ever consumed walnut, pecan, Brazil nut, and hazelnut but he consumed peanut, almond, pistachio and cashew without concern at least a few times a month. At that first visit he was assessed for Ig-E mediated allergies with skin prick testing. SPT demonstrated no reaction to walnut, pecan, hazelnut, and Brazil nut extracts. The family was advised to introduce these tree nuts into his diet and continue pistachio, cashew, hazelnut, peanut and almonds. Due to fear, his mom never introduced them at home, and he only consumed peanut and almonds.

At age 6, he was referred to our clinic for re-assessment of his walnut allergy. Skin prick testing to walnut extract was negative. IgE testing was also performed which showed walnut IgE < 0.35 kU/L, and serum IgE 58 µg/L. He was advised to introduce walnut at home along with all other tree nuts and to keep on eating peanut and almond.

Between the ages of 6 to 12, he experienced up to 4 episodes of emesis after consuming walnuts, all occurring within thirty to sixty minutes after ingestion. In retrospect, the reactions occurring less than 1 h may be attributed to recall bias. They did not require emergency care, and there were no cutaneous, cardiorespiratory, or other gastrointestinal signs and symptoms. His mom also stated that macadamia and pine nut may have caused similar but milder symptoms. Due to his various reactions, mom never introduced hazelnut, and pecan and stopped giving him cashew, pistachio and hazelnut.

At the age of 12, he was reassessed by our clinic for walnut and tree nut allergy. Due to negative skin prick testing to walnut, pecan, hazelnut, brazil and pine nuts an open OFC to walnut was suggested to confirm that he did not have an Ig-E mediated reaction and was in keeping with oral food challenges to rule-out immediate-type food allergy Oral food challenge for FPIES protocol was not performed. With repeated negative testing and with his history of developmental delay we wanted to ensure that he was not experiencing food aversion or mom was not experiencing anxiety. The oral challenge was preceded by repeat skin prick testing to fresh walnut which was again negative.

For the OFC, the patient consumed a cumulative 9 g of walnut ingested in increasing amounts over one hour. He was clinically well after 1 h and was discharged home. Two hours after ingestion, he began to experience repetitive vomiting. Mom noted him to be pale and lethargic. As he continued to have emesis, he was sent to emergency by ambulance. He did not have any cutaneous or cardiorespiratory signs and symptoms, nor did he have any diarrhea. His vital signs remained stable on assessments apart from tachycardia. He received ondansetron and antihistamine medications, as well as oral rehydration therapy, with good effect and was discharged home.

After discussion with his mom, due to his developmental concerns and our concern for developing food aversion, it was decided to avoid walnut, pecan, macadamia and pine nuts currently with annual follow-up to reassess when these tree nuts could be re-challenged. We advise mom to keep peanuts and almonds in his diet approximately 3 times per week. We discussed with mom the pros- and cons- of re-introducing cashew, pistachio and hazelnut. These nuts he had previously tolerated and never had a reaction to, but mom wanted to hold off from introducing these nuts currently. We reviewed the limitation concerning natural history and cross-reactivity in FPIES. We also reviewed the risk of an acute episode is dehydration and not anaphylaxis and the importance of hydration and the use of anti-emetics.

## Discussion and conclusions

FPIES is a relatively new syndrome with limited literature available on food triggers. FPIES is classically seen in patients in early infancy, with cow’s milk or soy as the culprit food. This case report describes FPIES with walnut as a food trigger. It is also uncommon in that the patient had a late onset of symptoms starting from age 3.

### Diagnosing FPIES

FPIES is a clinical diagnosis that is made after ruling out other causes of emesis and/or diarrhea. The differential diagnosis for emesis is broad and can include anaphylaxis, infectious gastroenteritis, gastrointestinal reflux disease, or celiac disease. Given the patient’s history of developmental delay, it is prudent to also consider inborn errors of metabolism as a potential cause of emesis. A key difference between FPIES and these alternate diagnoses is the timeline and association with a single food trigger.

The patient had experienced multiple episodes of delayed emesis between ages 3 and 12, each associated with walnut ingestion, and resolving within 1 day. He was tested with skin prick and serum IgE testing, which were negative for any suspected food triggers and ruled out IgE-mediated food allergy suggesting he probably was experiencing recurrent acute mild FPIES. Part of the delay and challenge in diagnosing FPIES in this case could be attributed to not having all the clinical details as these episodes were not promptly assessed in a clinic or hospital setting. The recurrent episodes of acute FPIES were also not severe enough to require emergency department assessment and treatment, further limiting the amount of clinical information available.

The diagnosis was made after the patient underwent an OFC and subsequently met the major criterion, as well as minor criteria of repeat episodes of vomiting after eating walnuts, lethargy, pallor, and emergency department visit.

Most diagnoses of FPIES can be made clinically based on these criteria. However, variations in clinical presentation and lack of specific confirmatory tests make this syndrome more difficult to diagnose. This can lead to misdiagnoses and unnecessary investigations or treatments [14]. Although skin prick and serum IgE testing are helpful in distinguishing from IgE mediated allergies, they cannot be relied upon in FPIES to identify food triggers. However, IgE testing can still be used to identify atypical FPIES, which is associated with a prolonged course to tolerance of the food trigger [11, 12]. The gold standard of diagnosing FPIES is with an OFC. However, consideration needs to be given to the risks associated with OFCs, acute FPIES can lead to a potentially severe reaction due to hemodynamic instability.

### Food triggers

Cow’s milk, soy, and grain are the most commonly reported food triggers, with there being geographical variations [10, 15]. Much less is known about other food triggers, as their incidence is much rarer. In a study of 462 FPIES cases, less than 1% were induced by treenuts [16].

In our case, the patient’s mom had concerns regarding treenut cross-reactivity. He had previously consumed peanut, almond, pistachio and cashew without concern, with the latter three being part of the treenut family. In the case of IgE-mediated food allergies, tree nuts as well as other food families such as fish and shellfish are commonly known to have cross-reactivity. However, there is a lack of data on the cross-reactivity of foods in FPIES. Most patients with FPIES will only have one food trigger. The risk of multiple concurrent food triggers in FPIES increases with solid food FPIES, with 64% of solid food FPIES patients reacting to > 1 food trigger [11]. The most commonly reported co-sensitization in FPIES was with cow’s milk and soy, with 16 to 66% of patients reacting to both [11, 12, 17, 18]. The mechanisms behind these co-sensitizations as well as the pathophysiology of FPIES remains to be understood.

FPIES is thought to be a non-IgE, T cell-mediated response that contributes to gastrointestinal inflammation [10, 12, 13, 19]. However, patients with atypical FPIES will have IgE specific to the food trigger. These patients were more likely to have a longer period of reactivity towards the food trigger and have also been observed to be at higher risk of converting to IgE-mediated food allergy [10, 12, 13].

### Natural history of FPIES

There is limited information describing the natural history of FPIES. The most amount of data exists on FPIES in infancy, as this is when the offending food is usually first introduced [2, 10, 11, 16].

Resolution of FPIES is determined by OFC. The majority of cow’s milk FPIES will resolve by age 2, and 85% of FPIES resolve by age 5 [2, 16]. However, there are cases of persistent FPIES that last into the teenage or adult years [16, 20]. Resolution of FPIES is typically prolonged with solid food triggers, older age of onset, and in atypical FPIES where there are food specific Ige’s [11, 12, 21].

Once patients have been diagnosed with FPIES, they are advised to avoid the trigger food until an OFC can be conducted to determine if the allergy still exists. During this period of avoidance, it is unknown if accidental re-exposures will have any effect on the time to resolution.

In this case, the patient had an initial presentation of likely FPIES at age 3. Given that the patient’s FPIES did not present during infancy, there is less consensus on when his FPIES can be expected to resolve. There are limited data that show resolution of grain, meat, and fish induced FPIES by age 5 in 65.5%, 50%, and 0% of patients respectively [11]. Limited data on resolution of FPIES in adolescents and adults show that even de novo presentations of FPIES later in life can spontaneously resolve with a widely variable duration [20]. This can be complicated by the fact that not all patients are willing to undergo OFC, and rather choose to continue to avoid the specific food.

### Follow-up planning

During assessment of the patient’s food allergy, his mom had concerns that he also had symptoms to pecan, macadamia, and pine nuts, which were negative on skin prick testing. OFC would be needed to make the diagnosis of FPIES. In discussion with the patient’s mom, it was decided that the OFC would be deferred, and the patient would continue to avoid walnuts, pecans, macadamia, and pine nuts. We also discussed the importance of re-introducing previously tolerated peanut, cashew, pistachio and hazelnut with mom. Repeat OFC can be performed in the future, should the patient wish to determine if his reaction has resolved.

Patients with FPIES should also be provided instructions on what to do if they develop another FPIES reaction. This can include information on when to seek emergency treatment, and treatment options that can be given at home if symptoms are mild. We also provided this to the patient’s family in the form of a letter that could be taken to the emergency department, so that treatment is not delayed for this potentially serious syndrome.

## Conclusion

This case describes acute FPIES triggered by walnut. There remains a lack of information on the natural history of FPIES, especially towards uncommon food triggers and on FPIES that present beyond infancy. As FPIES becomes a more recognizable syndrome, it would be beneficial especially with the early introduction of allergenic food guidelines for future reports to fill in gaps on other food triggers and natural history.

## Data Availability

Data sharing does not apply to this article as no datasets were generated or analyzed during the current study.

## Abbreviations

FPIES: Food protein-induced enterocolitis syndrome
OFC: Oral food challenge
